# Periodontal Diseases and the Risk of Metabolic Syndrome: An Updated Systematic Review and Meta-Analysis

**DOI:** 10.3389/fendo.2020.00336

**Published:** 2020-06-09

**Authors:** Romila Gobin, Dan Tian, Qiao Liu, Jianming Wang

**Affiliations:** ^1^Department of Epidemiology, Center for Global Health, School of Public Health, Nanjing Medical University, Nanjing, China; ^2^Department of Chronic Communicable Disease, Center for Disease Control and Prevention of Jiangsu Province, Nanjing, China

**Keywords:** periodontal disease, periodontitis, periodontal pocket, clinical attachment loss, metabolic syndrome, meta-analysis

## Abstract

**Background:** Periodontitis and metabolic syndrome (MetS) are two major global health problems that are widely prevalent in the world, although the former is a common infection in developing countries and the latter is a non-infectious but prevalent disease in developed countries. This study aims to provide an updated review on the existence and magnitude of the relationship between periodontal disease and the risk of MetS.

**Methods:** We searched the PubMed, Web of Science, ScienceDirect, Chinese National Knowledge Infrastructure, and Wanfang databases for original studies assessing the association between periodontitis and MetS published before August 2019. We calculated the pooled crude and adjusted odds ratios (ORs) together with the 95% confidence intervals (95% CIs) to estimate the strength of this association. Subgroup analysis was performed by considering the diagnostic method or the country where the studies were performed.

**Results:** We identified 43 potentially eligible articles for this systematic review, including 32 cross-sectional studies, eight case–control studies, and three cohort studies. Among them, 39 articles presented enough information to be included in the meta-analysis. The pooled crude and adjusted ORs were 1.99 (95% CI: 1.75–2.25) and 1.46 (95% CI: 1.31–1.61), respectively. Subgroup analysis showed a consistent relation stratified by either the diagnostic method or the country where the studies were performed. The pooled OR was 1.68 (95% CI: 1.41–2.00) for Japan, 1.75 (95% CI: 1.31–2.34) for the USA, 1.81 (95% CI: 1.35–2.42) for Korea, and 2.29 (95% CI: 1.53–3.41) for China.

**Conclusion:** Our results provide compelling evidence for the association between periodontitis and MetS. Patients with periodontal disease are a critical screening population for MetS. We also recommend that people exhibiting components of MetS should receive a periodontal check-up and pay attention to their oral health.

## Introduction

Oral health is an undervalued parameter of global health and has been considered inferior on the agendas of policymakers (1). There has been increasing scientific interest regarding the interactions between oral health and systemic diseases. In 2007, the World Health Organization (WHO) called on the integration of health policies regarding oral and general health (1). The European Union and the USA have also emphasized the importance of oral health in overall health (2). Numerous studies have shown a relationship between deficiently poor oral hygiene and different systemic disorders, such as cardiovascular disease (CVD) and metabolic syndrome (MetS). A variety of theories have been proposed, of which a bulk of them hypothesize the mediation of the inflammatory response (3). Periodontitis is one of the most common chronic oral diseases and is characterized by the pathological loss of the periodontal ligament and adjacent supporting alveolar bone. It can also be described as a bacteria-induced complex chronic inflammatory disease (4). It is estimated that approximately 20 to 60% of the world's population may have some degree of periodontal disease (5). Periodontitis is not merely a consequence of plaque accumulation; it is also affected by host factors (6). If discovered in the initial stage, it can be managed successfully without causing much morbidity.

MetS is a prevalent and multifactorial disorder that consists of a cluster of several clinical physical conditions and biological abnormalities that increase the risk of mortality; MetS is affected by insulin resistance and promotes cardiovascular diseases (7). These conditions/diseases include glucose intolerance/insulin resistance/hyperglycemia, hypertension, visceral obesity, and dyslipidemia (8). MetS has an estimated prevalence of 17–32% in the general population (9), which suggests that nearly one-quarter of adults throughout the world are affected (10).

The association between periodontitis and MetS has gained research interest in the scientific literature. There is variation in the reported degree of association, which may be due to the different definitions of MetS, the methodology used to assess periodontitis, and criteria for subject enrollment (11). In addition, there are several different approaches to determine the association between these two diseases/conditions. The American Academy of Periodontology (AAP) and the European Federation of Periodontology (EFP) have emphasized that more studies are needed to assess the association between periodontitis and various systemic conditions, including MetS (12). This study aims to provide an updated review on the existence and magnitude of the relationship between periodontal disease and the risk of MetS. A better understanding of periodontal diseases in the development of MetS and vice versa is required for medical and dental professionals to provide the general population with appropriate care.

## Methods

### Literature Search and Study Selection

The PRISMA (Preferred Reporting Items for Systematic Reviews and Meta-Analyses) guidelines were used (Supplementary Table 1) (13). We searched the PubMed, ScienceDirect, Web of Science (WOS), Chinese National Knowledge Infrastructure (CNKI), and Wanfang databases for studies that reported an association between periodontitis and MetS in adults up to August 2019. Studies were limited to human studies and those published in either English or Chinese. Related keywords used for PubMed were as follows: Periodontal disease OR periodontitis OR periodontal pocket OR clinical attachment loss AND metabolic syndrome OR metabolic disease OR syndrome X OR Reaven's syndrome OR MetS. PubMed MeSH terms were as follows: Metabolic Syndrome/Syndrome X (MeSH term Metabolic Syndrome X for both) and Periodontal Diseases, Periodontitis, Pocket Depth (No MeSH term), Periodontal Pocket, Periodontal Pocketing (MeSH term Periodontal Pocket), Attachment Loss (MeSH term Periodontal Attachment Loss), and Clinical Attachment Loss (MeSH term Tooth Mobility). Keywords for WOS and ScienceDirect searches were metabolic syndrome/periodontal disease, metabolic syndrome/periodontitis, metabolic syndrome/pocket depth, and metabolic syndrome/periodontal pocket. The terms we used were explored to ensure the retrieval of all the items needed concerning the specific search terms. Titles and abstracts of selected studies were examined for their possible relevance to the association between periodontitis and MetS. Studies were considered eligible using the following criteria: original epidemiological studies; observational studies including a cross-sectional, case–control, or cohort design; adult samples; having at least one diagnostic standard for periodontitis that was clearly defined; and having clear criteria for the diagnosis of MetS. Studies matching the eligibility criteria were considered for this systematic review. Abstracts and reviews were excluded; however, references in reviews were used for a supplementary literature search. In total, 43 studies (38 published in English and five published in Chinese) met the inclusion criteria for the systematic review with a total sample size of 114,181 participants. Among them, 39 studies had enough information to be included in the meta-analysis. A random-effect model was conducted to estimate the pooled odds ratios (ORs) together with 95% confidence intervals (95% CIs) to establish the strength of association.

### Data Extraction

Two reviewers independently identified eligible studies on the above topic, assessed the publication validity, and subsequently extracted data. The discrepancies were discussed and resolved, and the studies were concluded after consultation with the supervisor of this research. We collected data on the year of publication, the first author, study country, study design, sample size, mean age, sex, current tobacco smoking status, criteria for periodontitis diagnosis, periodontal examination protocol, the number of subjects diagnosed with periodontitis, criteria for MetS diagnosis, the number of subjects diagnosed with MetS, and the strength of the association [OR, relative risk (RR) and 95% CI].

### Quality Assessment

We used the Newcastle–Ottawa scale to evaluate the quality of the studies. A “star system” was used to judge the study in the following three contexts: the selection of the study groups, the comparability of the groups, and the ascertainment of either the exposure or the outcome of interest. The results varied across the selected studies, which is shown in Supplementary Table 1. The scale has a score of 0–9 stars for each article. A higher number of stars indicates a higher quality of the study (14).

### Statistical Analysis

The associations between periodontitis and MetS were assessed by the pooled ORs with the corresponding 95% CIs. Data from 39 studies were collected individually, and a crude OR was calculated, followed by the random-effect model. Then, the second analysis, consisting of the pooled adjusted OR, was derived from the given adjusted ORs from each study. *I*^2^ was also used to test the heterogeneity among the included studies. Consequently, subgroup analysis was first performed on the method used to diagnose periodontitis, that is, either partial-mouth periodontal examination or full-mouth periodontal examination. The second subgroup analysis was on the criteria for the diagnosis of MetS, which are the National Cholesterol Education Program Adult Treatment Panel III (NCEP ATP III) criteria, the 2005 International Diabetes Federation (IDF, 2005) criteria, or the 2009 IDF criteria. Finally, the subgroup analysis was performed by country, namely, China, Japan, the USA, and Korea, the countries where most of the published studies have been conducted. The subgroup analysis was performed using both the crude and the adjusted ORs separately. Stata version 15.0 (StataCorp, College Station, Texas, USA) was used to analyze the data.

## Results

### Description of Studies

**Figure 1:**
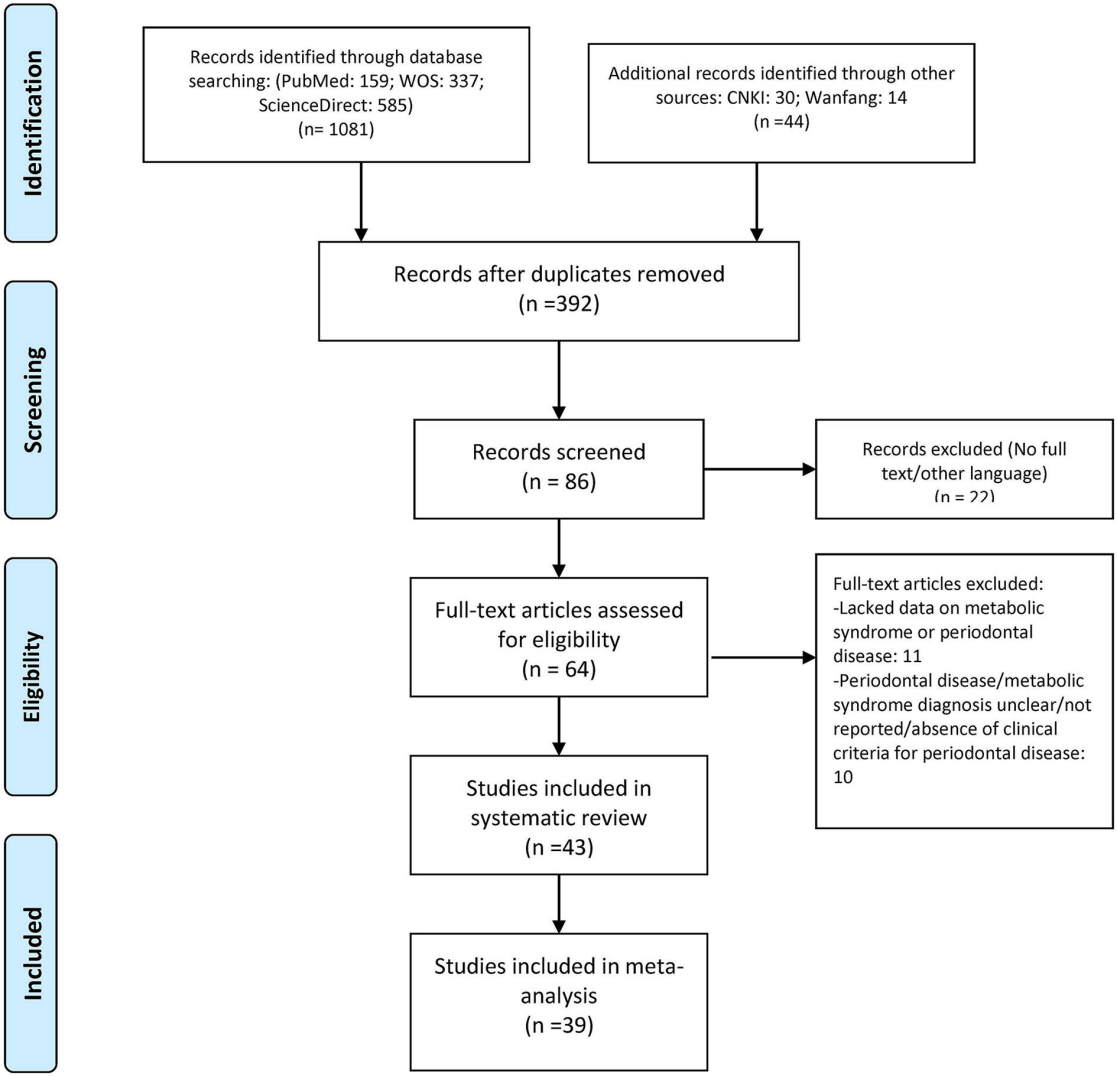
PRISMA flowchart of the search for studies and their selection and inclusion.

During the literature search, 1,125 articles were selected, and to find other associated studies, articles listed in their references were also screened. Duplicates and other non-related articles were not considered for the systematic review. Then, among these, 86 of the articles were retrieved, and their titles, abstracts, and full texts were scrutinized for possible relevance. Finally, 43 articles were selected for the systematic review, and 39 were enrolled in this meta-analysis where the crude ORs were calculated individually (Figure 1). Additionally, 35 studies with adjusted ORs were used from the specified studies (6, 10, 12, 15–54).

**Figure 2:**
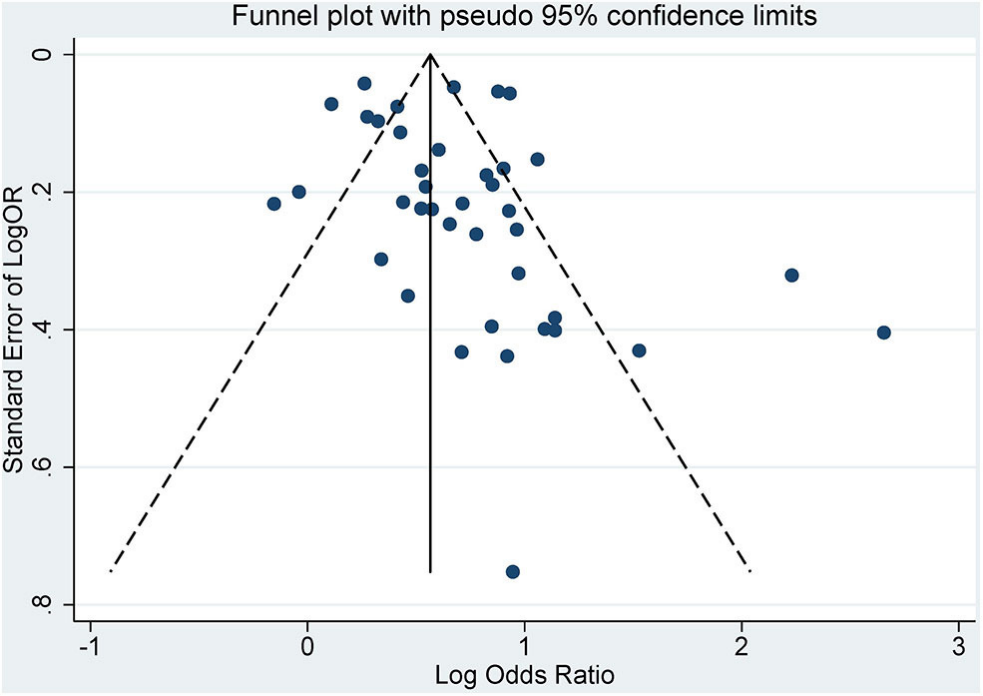
Funnel plot of included studies.

The characteristics of each study are listed in Table 1. Forty-three studies published from 2007 to 2019 were analyzed. There were studies from eastern Asia as well as western Asia, European countries, and North and South American countries. Thirty-eight were published in English, while 5 of them were published in Chinese. The sample size ranged from 120 (27) to 33,740. (33) Of the studies meeting the inclusion criteria for analysis, three were cohort studies (40, 45, 53), 32 were cross-sectional studies, and 8 were case–control studies. The criteria used for the diagnosis of periodontitis were different in these studies. Some used the CPI index, while others used the CAL or PPD. Twenty-seven studies had complete full-mouth examination, 15 had partial-mouth examination, and one study (28) was self-reported periodontitis. The proportion of periodontitis ranged from 3.4 to 88.4% among the mentioned studies. For the diagnosis of MetS, 21 studies used NCEP ATP III criteria, 5 used the 2005 IDF criteria, 13 used the 2009 IDF criteria, and the remaining studies used different methods. The percentage range of patients with MetS ranged from 5.2 to 100% among the studies. The funnel plot for the investigation of publication bias was drawn by plotting the standard error of the logOR against the logOR. As shown in the plot, the risk of publication bias was not significant (Figure 2).

**Table 1:** Description of included studies.

**Year**	**Author**	**Country**	**Study design**	**Sample size**	**Mean age (years)**	**Male (%)**	**Female (%)**	**Current smokers (%)**	**Criteria for periodontitis diagnosis**	**Periodontal examination protocol**	**Subjects with periodontitis (%)**	**Criteria for MetS diagnosis**	**Subjects with MetS (%)**	**Measures of association OR/RR (95% CI)**
2007	LI PENG	CHINA	CASE–CONTROL	501	61.4	40.6	59.4	23.2	CAL/PPD	COMPLETE	72.4	IDF (2005)	47.9	Association was not reported
2007	SHIMAZAKI Y.	JAPAN	CROSS-SECTIONAL	584	55.7	NIL	100	6.7	CAL/PPD	PARTIAL	(1) PPD: 17.1 (2) CAL: 6.3	NCEP ATP III	16.8	OR: (1) 6.6 (95% CI: 2.6–16.4); (2) 4.2 (95% CI: 1.2–14.8)
2008	D'AIUTO FRANCESCO	UNITED STATES	CROSS-SECTIONAL	13677	48	50.6	49.4	30.2	CAL/PPD	PARTIAL	14	IDF (2005)	20.3	OR: 1.45 (95% CI: 0.91–2.33)
2008	KHADER YOUSEF	JORDAN	CASE–CONTROL	156	47.2	35.9	64.1	41	CAL/PPD	COMPLETE	27.6	NCEP ATP III	50	The difference in the adjusted means between subjects with and without MetS was 2.2 mm for PD, 0.99 mm for CAL, 23.2% for the percentage of sites with CAL ≥3 mm, and 25.6% for the percentage of sites with PD ≥3 mm.
2009	LI PENG	CHINA	CASE–CONTROL	192	60.8	40.9	59.1	20.2	CAL	COMPLETE	72.4	IDF (2005)	72.9	OR: 15.6 (95% CI: 2.20–110.43)
2009	MORITA TOYOKO	JAPAN	CROSS-SECTIONAL	2478	43.3	81.8	18.2	32	CPI code 3–4	PARTIAL	25.9	JSIM/IDF (2005, MODIFIED JAPANESE)	8.2	OR: 2.4 (95% CI: 1.7–2.7)
2009	KUSHIYAMA MITOSHI	JAPAN	CROSS-SECTIONAL	1070	55	26.3	73.7	9.3	CPI code 3–4	COMPLETE	29.5	NCEP ATP III	9.1	OR: 2.13 (95% CI: 1.22–3.70)
2010	ANDRIANKAJA OM	UNITED STATES	CROSS-SECTIONAL	7431	40.1	47.3	52.7	26.8	PPD	PARTIAL	5.8	NCEP ATP III	19.7	MEN—OR: 1.0 (95% CI: 0.7–1.6) WOMEN—OR: 2.1 (95% CI: 1.2–3.7)
2010	NESBITT MARK J.	UNITED STATES	CROSS-SECTIONAL	200	56.8	57.8	42.5	57.5	Radiographic distance CEJ-crestal bone ≥ 3 mm	COMPLETE	21.5	NCEP ATP III	17.5	OR: 2.61 (95% CI: 1.1–6.1)
2010	BENGUIGUI CATHERINE	FRANCE	CROSS-SECTIONAL	255	58	54.9	45.1	19.2	Page & Eke 2007	COMPLETE	78.8	NCEP ATP III	28.6	OR: 3.97 (95% CI: 1.22–12.9)
2010	HAN D-H	SOUTH KOREA	CROSS-SECTIONAL	1046	42.3	43.7	56.3	26.5	CPI CODE 3–4	PARTIAL	34	IDF (2009)	22.4	OR: 1.70 (1.22–2.37)
2010	TIMONEN P.	FINLAND	CROSS-SECTIONAL	2050	47	39.3	60.7	NIL	PPD	COMPLETE	3.4	EGIR (2002)	16.4	Pockets ≥ 4 mm—RR: 1.19 (95% CI: 1.01–1.42) Pockets ≥ 6 mm—RR: 1.50 (95% CI: 0.96–2.36)
2010	ZHANG JIAN-QUAN	CHINA	CROSS-SECTIONAL	120	53	41.7	58.3	24.2	CAL/PPD	PARTIAL	85.8	CHINESE INTERNAL MEDICINE PROTOCOL	100	Not specified
2011	BENSLEY LILIAN	UNITED STATES	CROSS-SECTIONAL	481	48	41.5	58.5	21.7	SELF-REPORTED	NOT APPLICABLE	45.1	AHA (2009)	35.6	Adjusted complete case analysis, participants with severe periodontal disease were 1.5 times more likely to have metabolic syndrome
2011	KWON YOUNG-EUN	KOREA	CROSS-SECTIONAL	6520	46.4	39.5	60.5	39.1	CPI CODE 3–4	PARTIAL	45.6	NCEP ATP III	28.4	OR: 1.55 (1.32–1.83)
2011	CHEN LI-PING	TAIWAN	CROSS-SECTIONAL	253	58.8	46.2	53.8	29.6	No periodontitis: PDI score of 0–3, mild periodontitis: 3 < PDI score ≤ 4 moderate-to-severe periodontitis: 4 < PDI score ≤ 6	PARTIAL	58.9	NCEP ATP III	57.3	OR: 2.73 (1.29–5.79)
2012	HAN D-H	KOREA	CASE–CONTROL	332	49.5	56.6	43.4	31.9	CPI CODE 3-4	PARTIAL	41.9	IDF (2009)	50	OR: 1.76 (95% CI: 1.06–2.93)
2012	FUKUI NAO	JAPAN	CROSS-SECTIONAL	6421	44.5	77	23	25.2	PPD	COMPLETE	25.5	NCEP ATP III	14.9	OR: 1.35 (95% CI: 1.03–1.77)
2012	YU Z.R.	CHINA	CROSS-SECTIONAL	903	62.6	50.5	49.5	20.2	CAL/PPD	COMPLETE	88.4	JOINT INTERIM STATEMENT/IDF (2009)	69.7	OR: 1.524 (95% CI: 1.066–2.328)
2013	TU YU-KANG	TAIWAN	CROSS-SECTIONAL	33740	49.8	45.3	54.7	NOT SPECIFIED	At least ONE tooth with periodontitis	PARTIAL	30.8	NCEP ATP III	NOT SPECIFIED	Females—OR: 1.52 (95% CI: 1.41–1.63) Males—OR: 1.04 (95% CI: 0.96–1.12)
2013	SORA NICOLETA	USA	CROSS-SECTIONAL	283	55.3	24	76	14.8	CAL/PPD	COMPLETE	10.9	NCEP ATP III	85.9	RR: 2.77 (95% CI: 1.11–6.93);
2013	FURATA MICHIKO	JAPAN	CROSS-SECTIONAL	2370	59.5	43.9	56.1	26.3	NHANES III	COMPLETE	33.2	JOINT INTERIM STATEMENT/IDF (2009)	35.1	Women—OR: 3.60 (1.30–12.61) Men—OR: 1.21 (0.59–2.49)
2014	LAMONTE MICHAEL J.	UNITED STATES	CROSS-SECTIONAL	657	65.5	NIL	100	2.1	Osteo-periodontitis	COMPLETE	72.6	NCEP ATP III	25.6	OR: 1.11 (95% CI: 0.71–1.75)
2014	THANAKUN SUPANEE	THAILAND	CROSS-SECTIONAL	125	47	42.4	57.6	8.8	1) AAP, 2) PD ≥4 mm	COMPLETE	46.4	IDF (2009)	64.8	OR: 3.60 (95% CI: 1.34–9.65)
2015	ALHABASHNEH ROLA	JORDAN	CROSS-SECTIONAL	280	53.8	50.7	49.3	21.8	CAL/PPD	COMPLETE	39.6	IDF (2005)	83.2	OR: 3.28 (95% CI: 1.30–8.30)
2015	MINAGAWA K.	JAPAN	CROSS-SECTIONAL	234	80	47.4	52.6	5.6	AAP/CDC (MODIFIED)	COMPLETE	77.4	IDF (2005, MODIFIED JAPANESE)	24.4	OR: 2.10 (95% CI: 1.03–4.28)
2015	IWASAKI MANASORI	JAPAN	COHORT	125	70	44	56	39.2	CAL	COMPLETE	NOT SPECIFIED	NCEP ATP III	21.6	RR: 2.58 (95% CI: 1.17–5.67)
2016	CHEN X.	CHINA	CROSS-SECTIONAL	303	34.9	100	NIL	31	CPI	COMPLETE	23.1	JOINT INTERIM STATEMENTS/IDF (2009, MODIFIED CHINESE)	38.3	OR: 3.378 (95% CI: 1.889–5.924)
2016	JARAMILLO ADRIANA	COLUMBIA	CASE–CONTROL	651	46.5	36.1	63.9	20.1	CAL/PPD	COMPLETE	66.2	AACE (2003)	5.2	OR: 2.72 (95% CI: 1.09–6.79)
2016	KUMAR NARESH	INDIA	CASE-CONTROL	259	38.5	52.9	47.1	13.1	CAL	COMPLETE	50.2	NCEP ATP III	22	OR: 2.64 (95% CI: 1.36–5.18)
2016	GOMES-FILHO ISAAC SUZART	BRAZIL	CROSS-SECTIONAL	419	59	38.2	61.8	29.8	CAL/PPD	COMPLETE	34.6	NCEP ATP III/IDF (2005)	NCEP: 60.9 IDF: 67.1	OR: 2.11 (95% CI: 1.01–4.40)
2016	KAYE E.K.	UNITED STATES	COHORT	751	61	100	NIL	3	CAL/PPD	COMPLETE	25.4	IDF (2009)/NCEP ATP III	IDF: 44.2 NCEPATP III: 37	PPD ≥ 5 mm—OR: 1.37 (1.14–1.65) CAL ≥5 mm—OR: 1.19 (1.00–1.41)
2016	MUSSKOPF MARTA L.	BRAZIL	CROSS-SECTIONAL	363	47.5	36.1	63.9	44.1	CAL/PPD	COMPLETE	26.9	IDF (2009)	54.8	PREVALENCE RATIO (PR): 1.17 (95% CI: 0.83–1.65)
2016	WU WEI	CHINA	CROSS-SECTIONAL	1000	28-58	89.5	10.5	60	CAL/PPD	COMPLETE	75.6	CHINESE INTERNAL MEDICINE PROTOCOL	39.7	The risk of periodontitis in obese people is 4.6 times higher than that in normal-weight people of the same age group
2017	ZUK ALEKSANDRA	CANADA	CROSS-SECTIONAL	1383	49	50.5	49.5	18.1	CAL	PARTIAL	16.2	AHA/NHLBI	15.3	OR: 1.28 (95% CI: 0.68–2.40)
2017	KIKUI MIKI	JAPAN	CROSS-SECTIONAL	1856	66.4	41.6	58.4	19.3	CPI	PARTIAL	50.3	JOINT INTERIM STATEMENT/IDF (2009)	36.4	OR: 1.89 (1.31–2.73)
2017	ZHANG LI	CHINA	CROSS-SECTIONAL	1415	39.9	50	50	6.7	CPI	COMPLETE	39.7	IDF (2009)	18.3	OR: 1.263 (95% CI: 1.079–1.479)
2018	KIM O.S	KOREA	CROSS-SECTIONAL	5078	64.7	41.6	58.4	10.7	CDC/AAP	PARTIAL	CDC/AAP: 81.2	IDF (2009)	48.7	MEN—RR: 1.43 (95% CI: 1.17–1.73)
2018	PHAM ANH VU THUY	VIETNAM	CASE–CONTROL	412	57.8	27.7	72.3	9	CDC/AAP (2012)	COMPLETE	28.6	JOINT INTERIM STATEMENT/IDF (2009)	50	OR: 4.06 (95% CI: 2.11–7.84)
2018	KOO HO SEOK	KOREA	CASE–CONTROL	10340	57.2	51.8	48.2	21	CPI CODE 3–4	PARTIAL	51.4	NCEP ATP III	33	OR: 1.12 (95% CI: 1.01–1.24)
2018	NASCIMENTO GUSTAVO G.	BRAZIL	COHORT	539	31	50.6	49.4	NOT SPECIFIED	AAP/CDC	COMPLETE	14.3	NCEP ATP III	13.3	RMSEA: 0.07 (95% CI: 0.05–0.09)
2019	ABDALLA-ASLAN RAGDA	ISRAEL	CROSS-SECTIONAL	470	55.8	45.8	54.2	38.1	AAP	COMPLETE	75.3	NCEP ATP III	37.4	OR: 14.28 (95% CI: 6.66–31.25)
2019	KIM JI-SOO	KOREA	CROSS-SECTIONAL	8314	55	46.4	53.6	19.6	CPI	PARTIAL	37.3	NCEP ATP III	34.1	OR: 1.42 (95% CI: 1.26–1.61)

### Meta-Analysis

**Figure 3:**
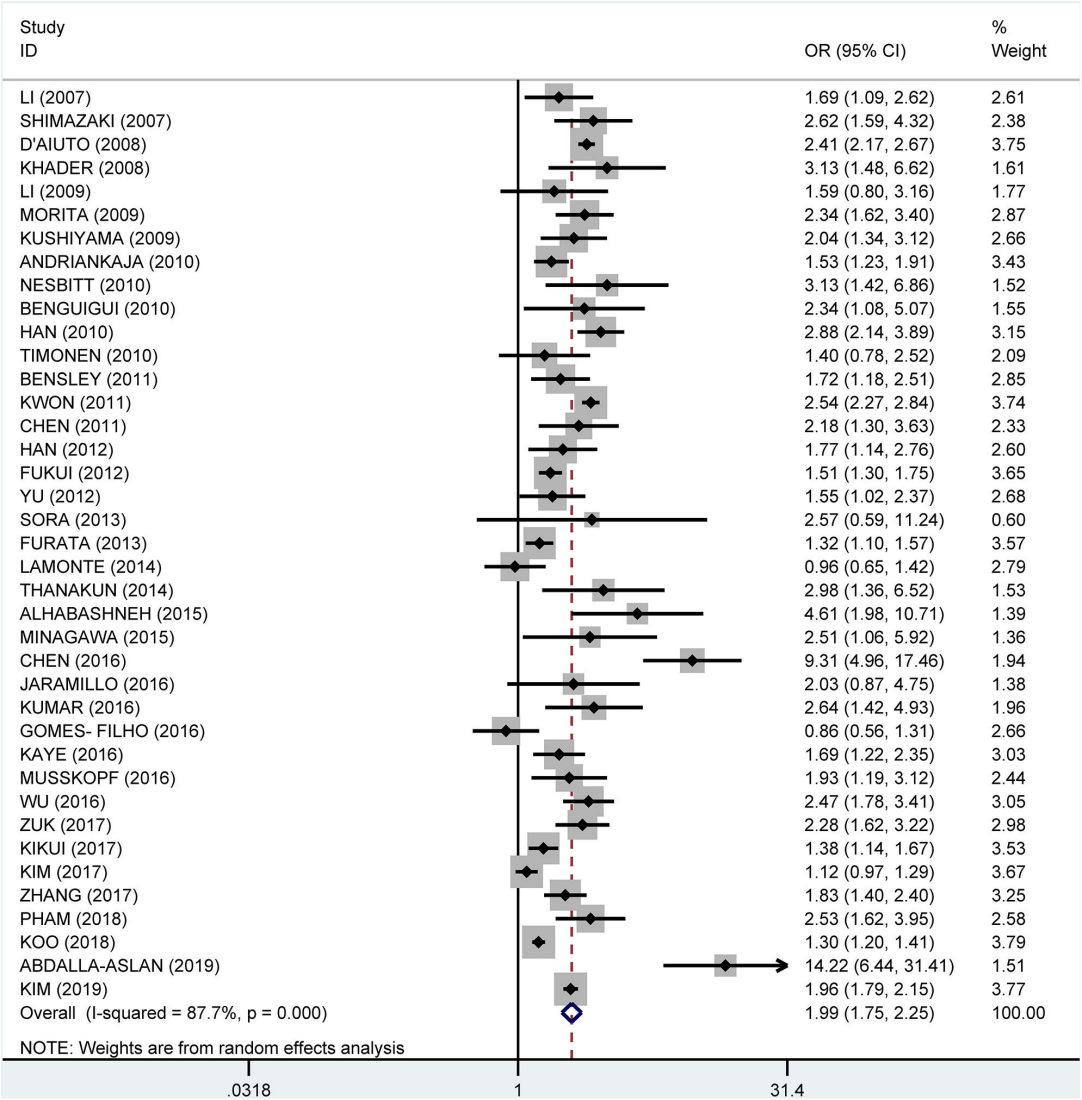
Pooled crude odds ratios of the association between periodontitis and metabolic syndrome.

**Figure 4:**
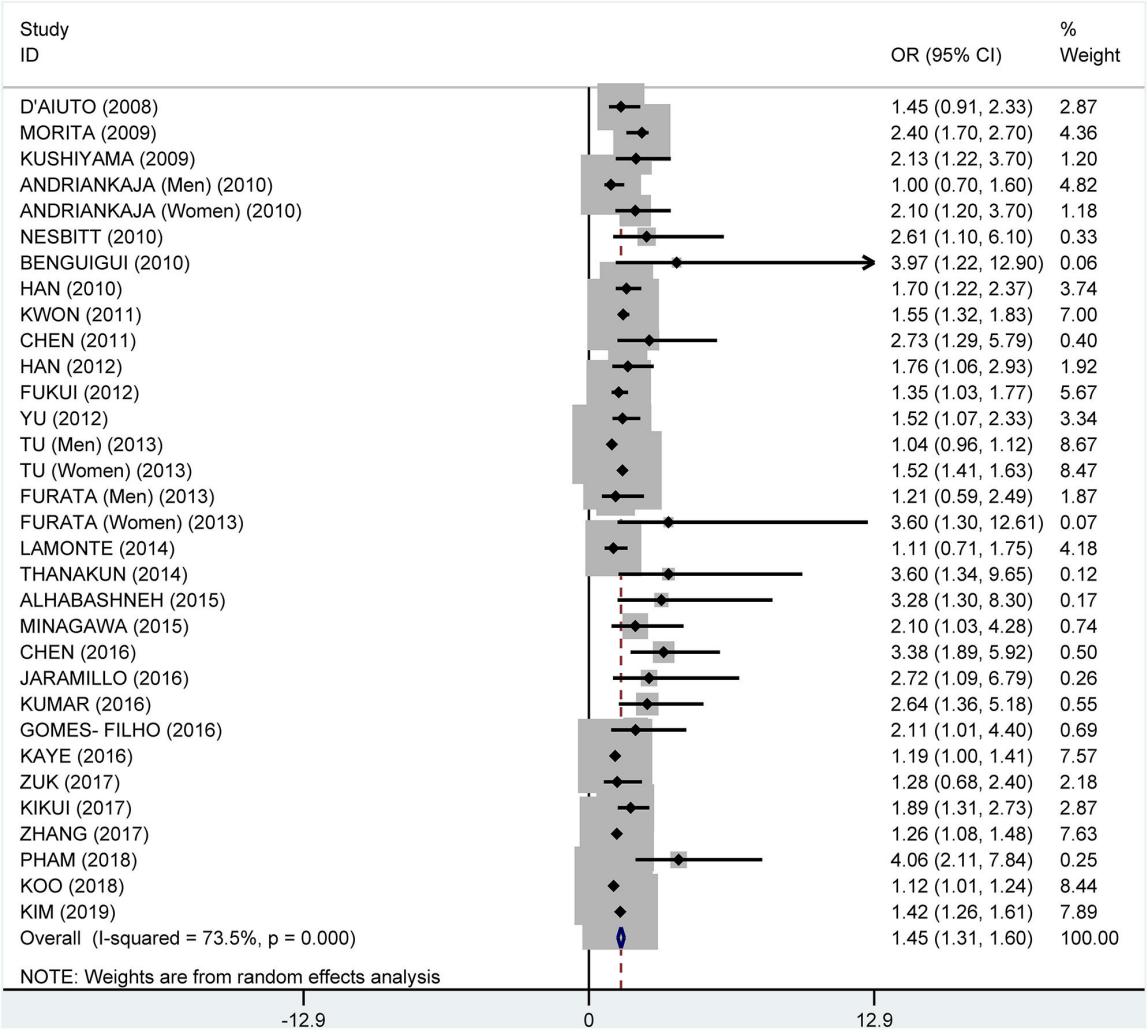
Pooled adjusted odds ratios of the association between periodontitis and metabolic syndrome.

From the data extracted, 39 of the studies showed an association of MetS and periodontitis, with a crude pooled OR of 1.99 (95% CI: 1.75–2.25). The heterogeneity test showed that *I*^2^ = 87.7% and *P* < 0.001 (Figure 3). We further summarized the adjusted ORs, which were mentioned in 32 studies, and showed a pooled adjusted OR of 1.45 (CI: 1.31–1.60). The heterogeneity test showed that *I*^2^ = 73.5% and *P* < 0.001 (Figure 4).

### Subgroup Analysis

**Figure 5:**
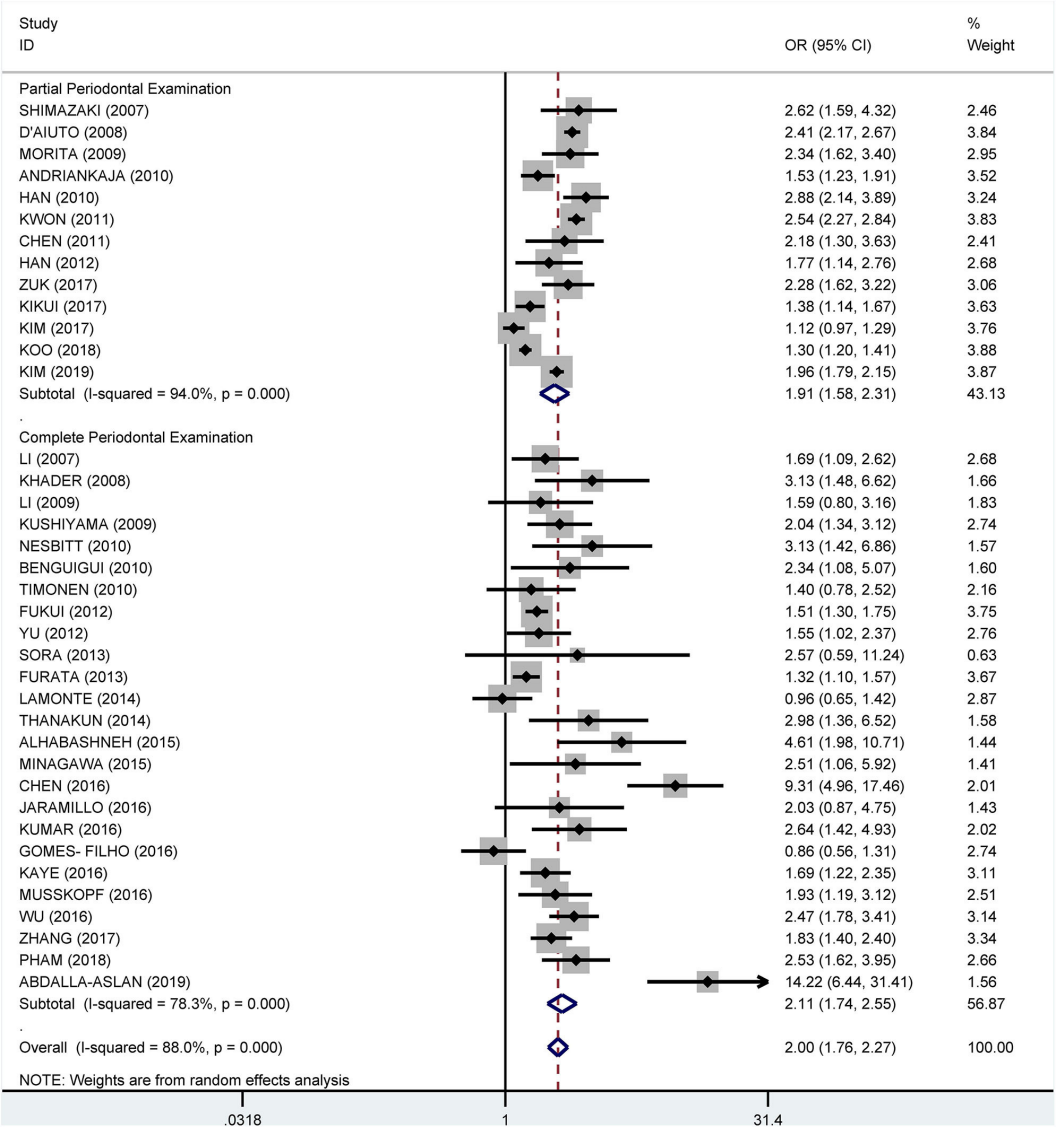
Subgroup analysis of pooled crude odds ratios of the association between periodontitis and metabolic syndrome by the method of examination used to diagnose periodontitis.

**Figure 6:**
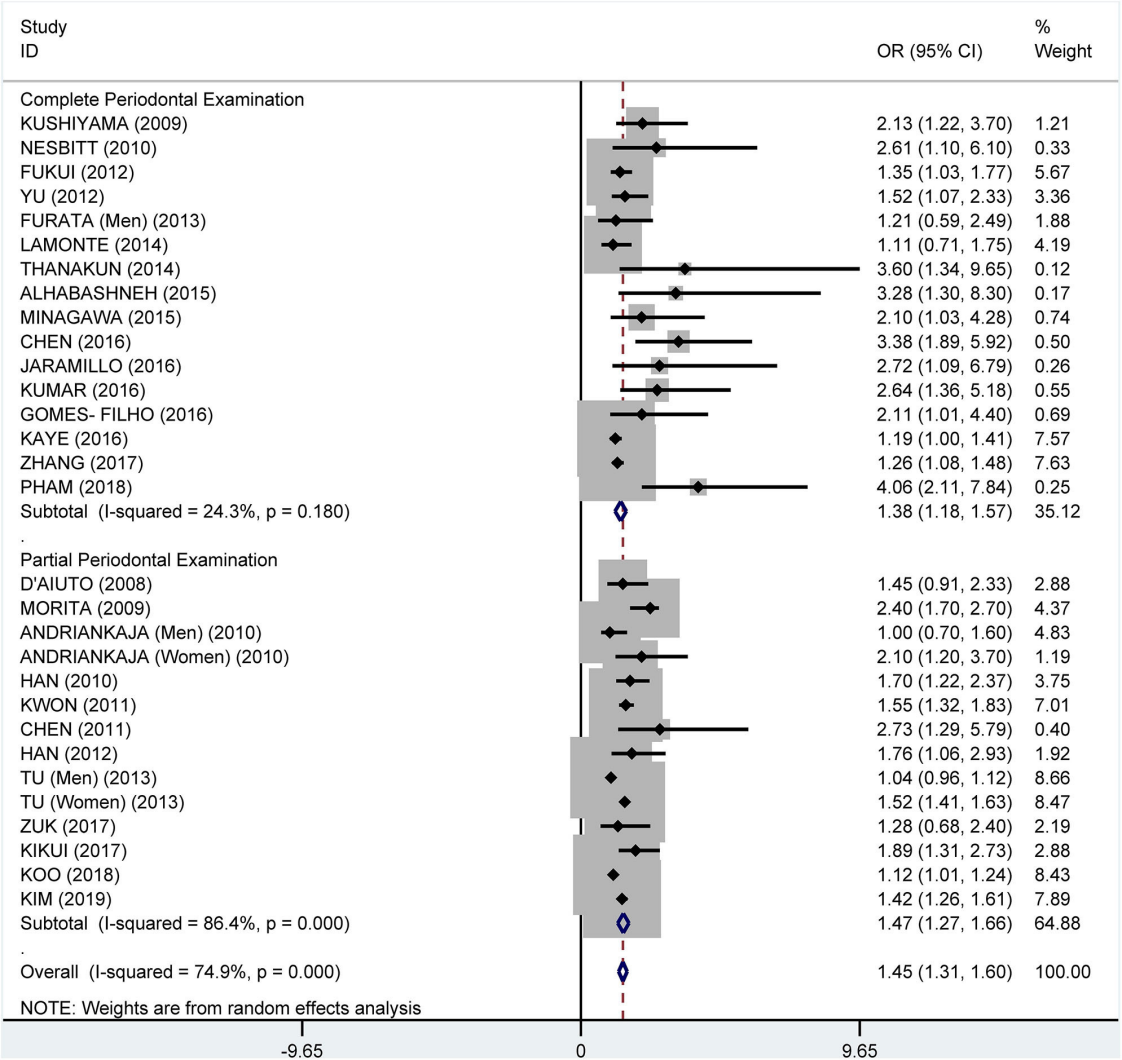
Subgroup analysis of pooled adjusted odds ratios of the association between periodontitis and metabolic syndrome by the method of examination used to diagnose periodontitis.

Subgroup analysis on the tooth examination used to diagnose periodontitis showed a crude OR of 1.91 (95% CI: 1.58–2.31, *I*^2^ = 94.0%, *P* < 0.001) for partial periodontal examination and a crude OR of 2.11 (95% CI: 1.74–2.55, *I*^2^ = 78.3%, *P* < 0.001) for complete periodontal examination (Figure 5). The pooled adjusted OR was 1.38 (95% CI: 1.18, 1.57), *I*^2^ = 24.3%, *P* = 0.180) for the complete periodontal examination and 1.47 (95% CI: 1.27–1.66, *I*^2^ = 86.4%, *P* < 0.001) for the partial periodontal examination (Figure 6).

**Figure 7:**
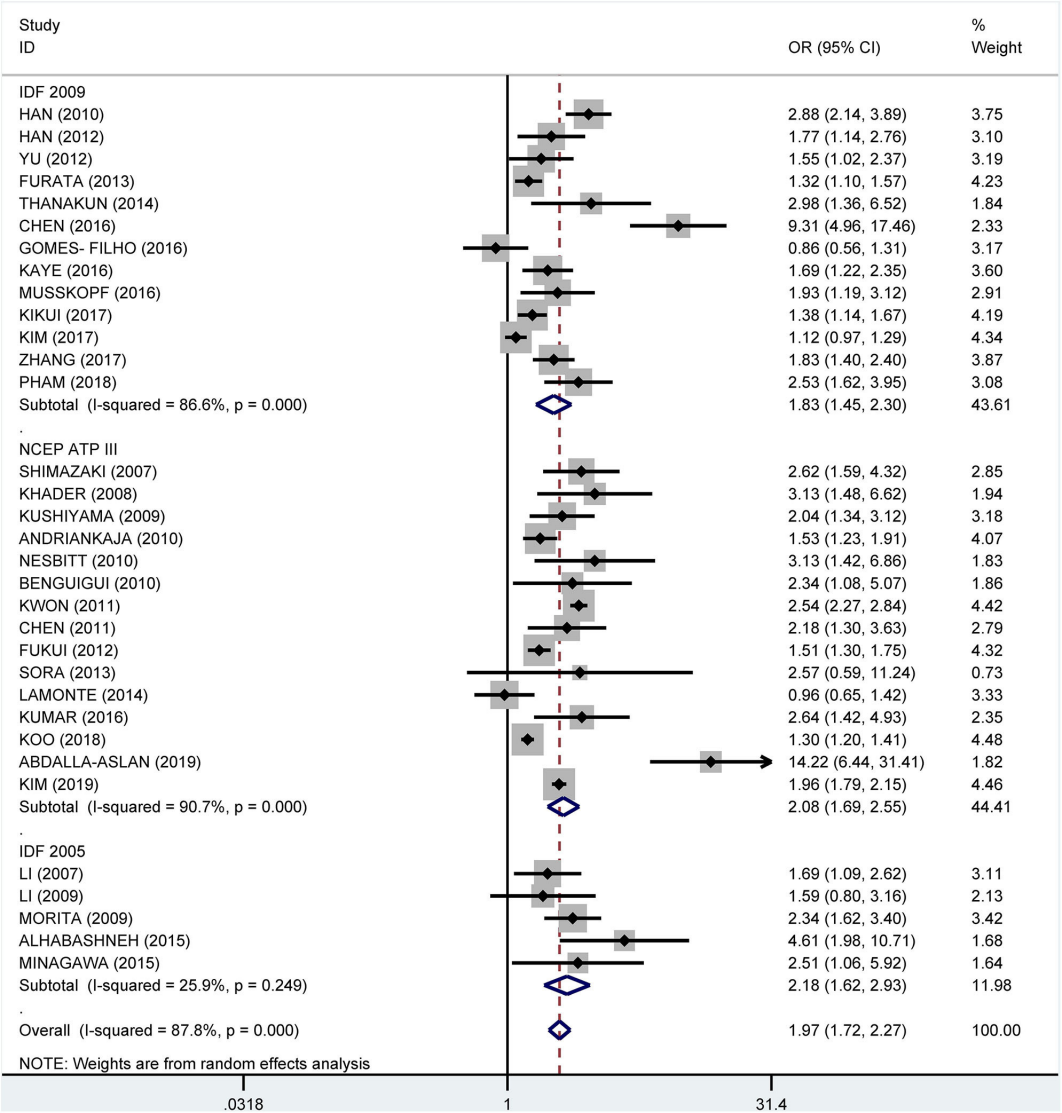
Subgroup analysis of pooled crude odds ratios of the association between periodontitis and metabolic syndrome by diagnostic criteria for metabolic syndrome.

**Figure 8:**
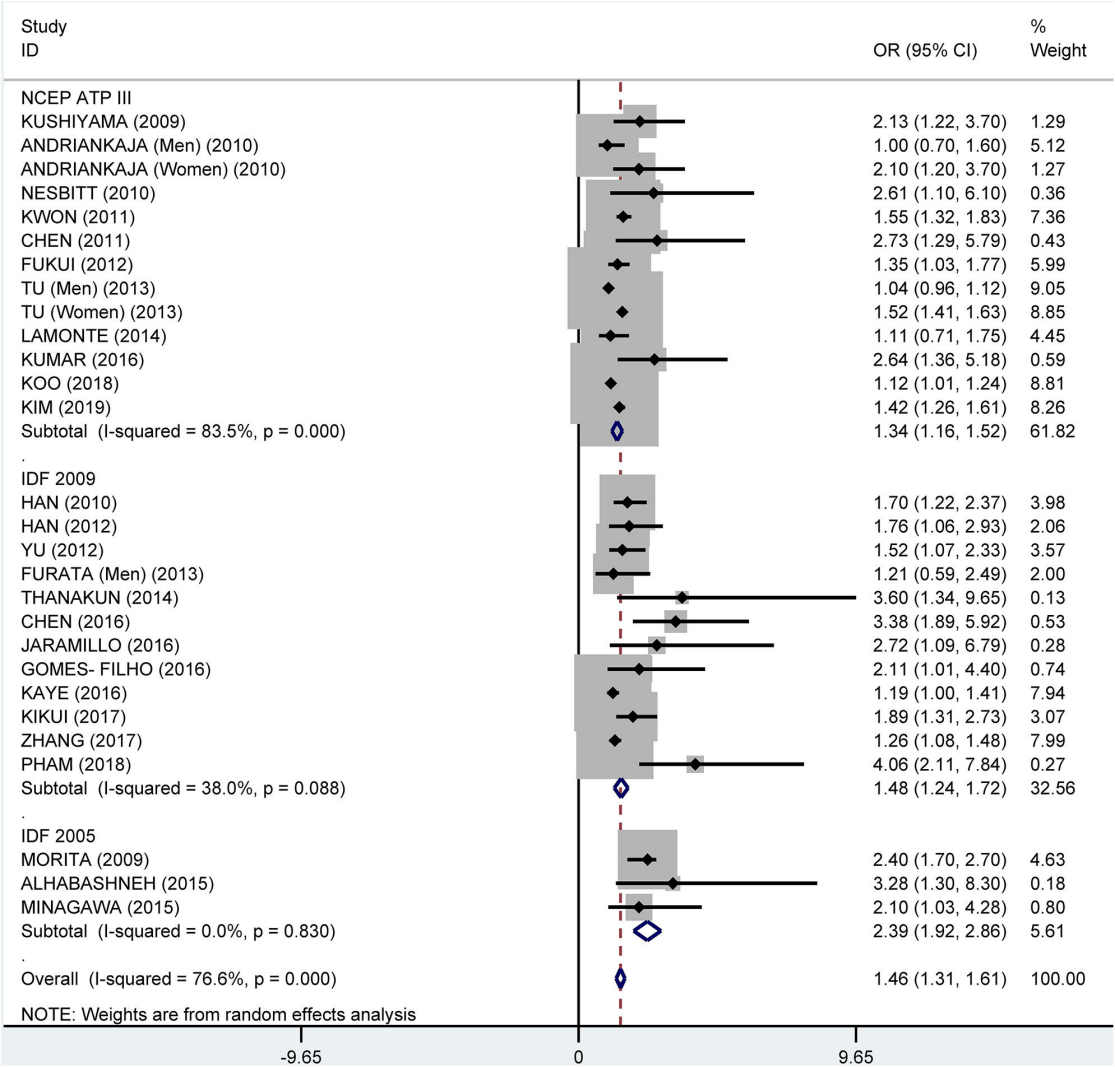
Subgroup analysis of pooled adjusted odds ratios of the association between periodontitis and metabolic syndrome by diagnostic criteria for metabolic syndrome.

Subgroup analysis by the diagnostic criteria of MetS showed that the crude OR was 1.83 (95%: 1.45–2.30, *I*^2^ = 86.6%, *P* < 0.001) for the 2009 IDF criteria, 2.08 (95% CI: 1.69-2.55, *I*^2^ = 90.7%, *P* < 0.001) for the NCEP ATP III criteria, and 2.18 (95% CI: 1.62–2.93, *I*^2^ = 25.9%, *P* = 0.249) for 2005 IDF criteria (Figure 7). The pooled adjusted OR was 1.34 (95% CI: 1.16, 1.52, *I*^2^ = 83.5%, *P* < 0.001) for the NCEP ATP III criteria, 1.48 (95% CI: 1.24–1.72, *I*^2^ = 38.0%, *P* = 0.088) for the 2009 IDF criteria, and 2.39 (95% CI: 1.92–2.86, *I*^2^ = 0.0%, *P* = 0.830) for the 2005 IDF criteria (Figure 8).

**Figure 9:**
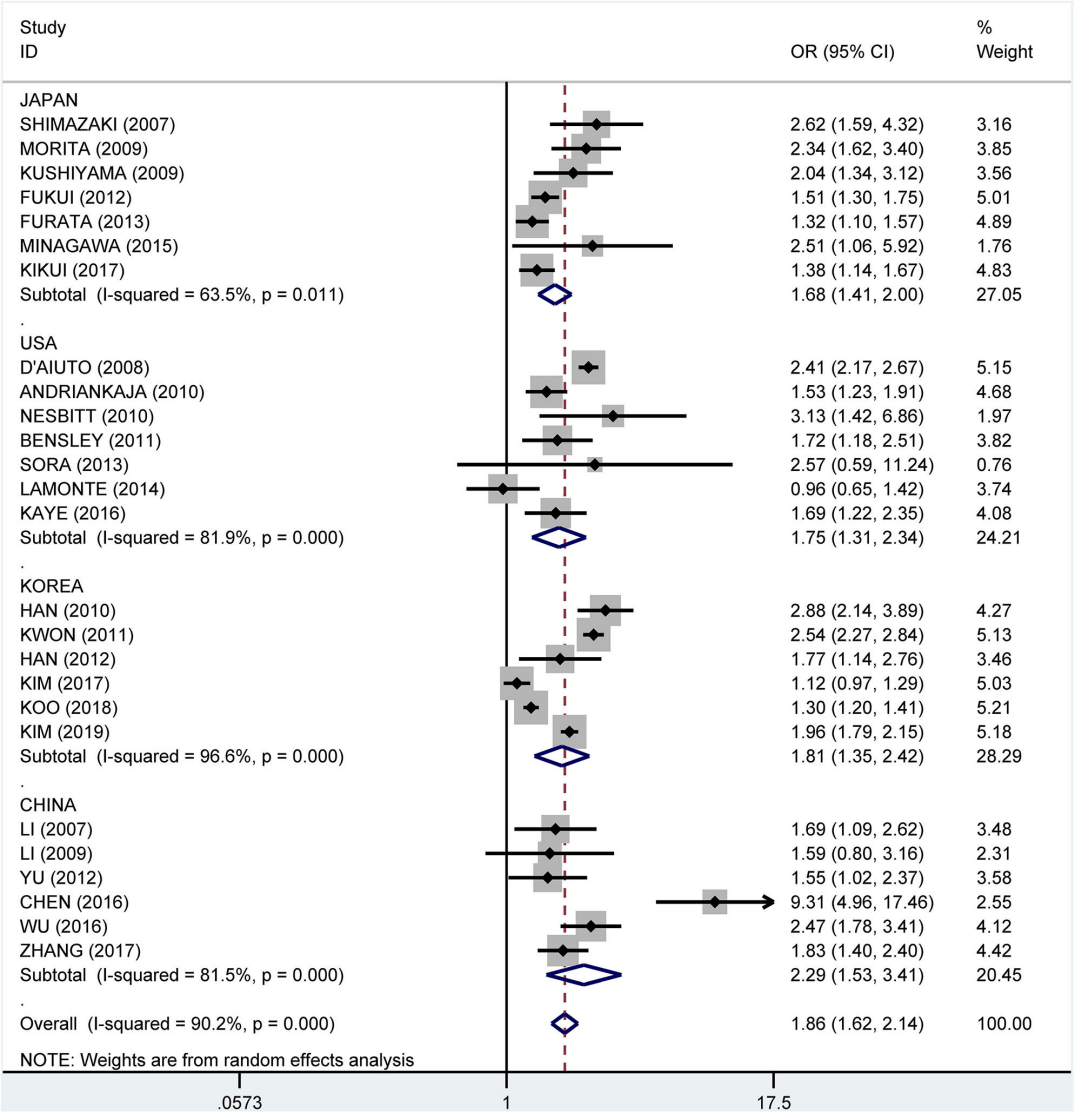
Subgroup analysis of pooled crude odds ratios of the association between periodontitis and metabolic syndrome by country.

**Figure 10:**
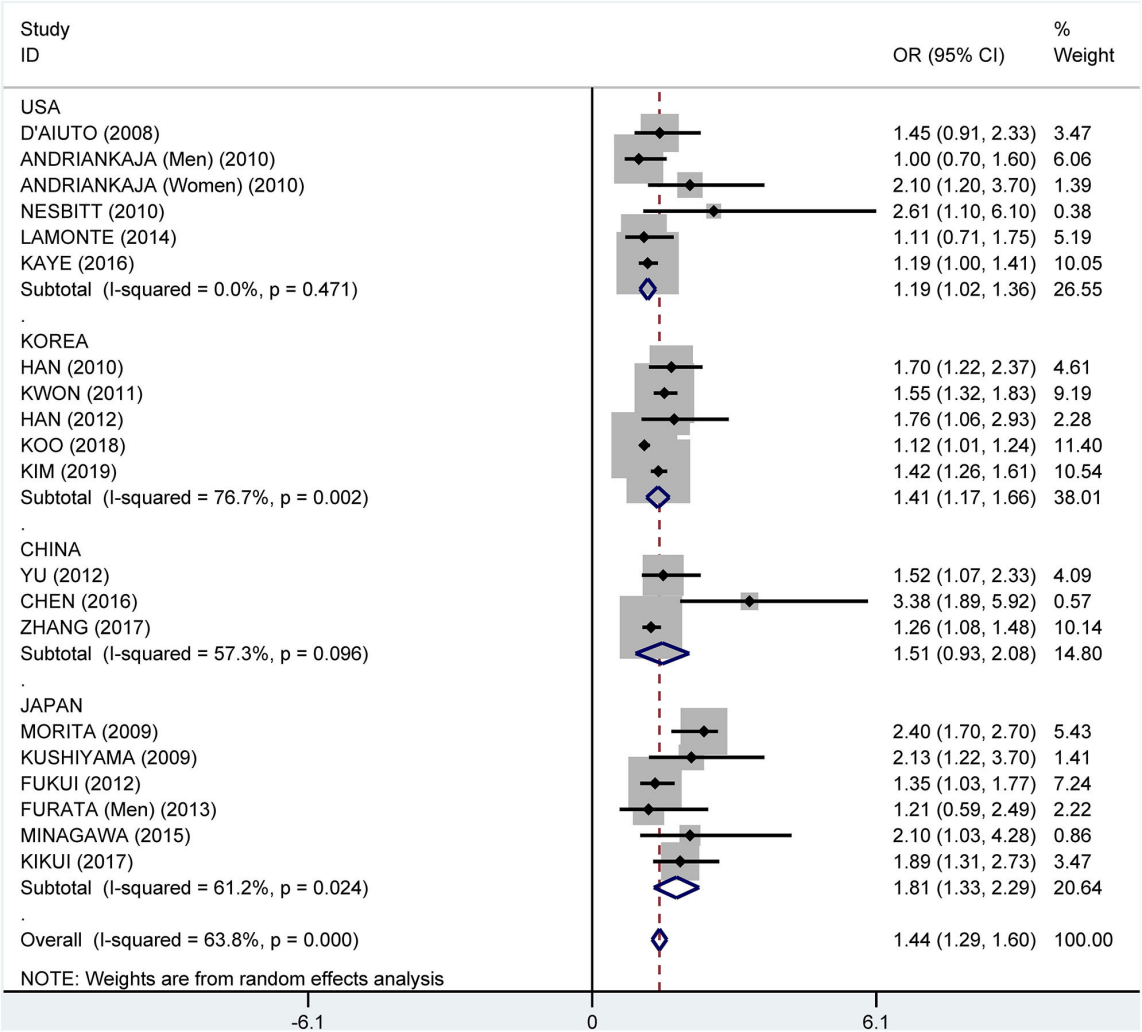
Subgroup analysis of pooled adjusted odds ratios of the association between periodontitis and metabolic syndrome by country.

Subgroup analysis by country showed crude ORs of 1.68 (95% CI: 1.41–2.00, *I*^2^ = 63.5%, *P* = 0.011) for Japan, 1.75 (95% CI: 1.31–2.34, *I*^2^ = 81.9%, *P* < 0.001) for the USA, 1.81 (95% CI: 1.35–2.42, *I*^2^ = 96.6%, *P* < 0.001) for Korea, and 2.29 (95% CI: 1.53–3.41, *I*^2^ = 81.5%, *P* < 0.001) for China (Figure 9). The adjusted OR was 1.19 (95% CI: 1.02–1.36, *I*^2^ = 0.0%, *P* = 0.471) for the USA, 1.41 (95% CI: 1.17–1.66, *I*^2^ = 76.7%, *P* = 0.002) for Korea, 1.51 (95% CI: 0.93–2.08, *I*^2^ = 57.3%, *P* = 0.096) for China, and 1.81 (95% CI: 1.33–2.29, *I*^2^ = 61.2%, *P* = 0.024) for Japan, respectively (Figure 10).

## Discussion

There is undoubtedly a positive association between periodontitis and MetS based upon the studies that were reviewed in this meta-analysis. There are a multitude of variables that can have ramifications in the association between periodontitis and MetS, including study population/subjects as well as ethnicity/geography, which have not been studied. Studies involved in this systematic review were conducted in 15 different countries worldwide, and the majority of the studies demonstrated a higher risk of MetS for patients with periodontal diseases.

The human body has multiple organ systems, and these systems are intimately and mutually dependent on each other. As a result, ailments in some organs or their components may play a contributing factor in the occurrence and evolution of a particular disease in other body locations (55, 56). The influence of oral health on one's general health has been scrutinized for the last decennium by a multitude of surveys (57). A systematic review including 72 studies and data from 291,170 individuals in 37 countries estimated that the global prevalence of severe periodontitis in 2010 was 10.8% (95% CI: 10.1–11.6%), thus making severe periodontitis rank 6th in the Global Burden of Disease (58).

The observation of a relationship between periodontitis and systemic diseases may significantly expand the scope and appreciation of oral health and dental practice. The general population, as well as non-dental medical professionals, should be informed that periodontal diseases play an important role in overall health. Due to a possible etiopathogenic relationship between periodontitis and various systemic diseases, patients with severe periodontal disease should be referred to screen for CVD, diabetes, MetS, and other systemic diseases. Patients prone to or diagnosed with certain systemic diseases may also be referred to dentists to check for and treat periodontal disease. Oral inflammatory lesions have different mechanisms concerning the possible association with systemic diseases (59–62). The oral cavity hosts several cell populations expressing mesenchymal stem cell like-features (63). The human periapical cyst mesenchymal stem cells (hPCy-MSCs) collected from the surgically removed periapical cysts exhibit interesting and valuable potentialities that could be of high impact in the future regenerative medicine applications (63). It is necessary to strengthen the cooperation between different clinical departments and treat the disease with a holistic view.

The main limitation of this particular review is that the analysis and comparison of ORs using the data available may be debatable, as the percentage of patients considered to have been diagnosed with periodontitis and MetS was determined with different diagnostic criteria. The fact that there is no global consensus on one specific set of diagnostic criteria for these two diseases is an enigma. Different researchers use different criteria depending upon the country where the study is being conducted, availability, and the accessibility of resources as well as specialized human resources to conduct the examination part of the survey, and this may affect the results of each study. Furthermore, most of the studies reviewed on this related issue are cross-sectional studies, and they do demonstrate an association of periodontal disease with MetS, but the prevalence ferreted at a single point thereby gives only a transient excerpt or cause–effect cannot be determined. An additional consideration is that many of the definitions of MetS allow the inclusion of subjects who have already been on medication for the treatment of some components of MetS, for instance, hypertension, diabetes, or dyslipidemia. The consequence of these medications on systemic inflammation is concealed, and some might have anti-inflammatory actions; thus, patients might have different periodontal statuses. One study reported by Shimazaki et al. showed that MetS increased the risk of periodontitis, with ORs for greater pocket depth and clinical attachment loss exhibited in 4 or 5 components of 6.6 (95% CI: 2.6–16.4) and 4.2 (95% CI: 1.2–14.8), respectively (16). Due to the lack of longitudinal studies, it is difficult to determine the relative contribution of periodontitis to MetS and the contribution of MetS to periodontitis. However, this does not affect our suggestion that clinicians and the general public should pay attention to the relationship between oral health and systemic disease.

## Conclusion

Our results provide compelling evidence for the association between periodontitis and MetS. Patients with periodontal disease are a critical screening population for MetS. We also recommend that people exhibiting components of MetS should receive a periodontal check-up and pay attention to their oral health. Among the golden rules for maintaining good oral hygiene is that one should visit a dentist at least twice a year, brush at least twice daily, floss regularly, and have a healthy diet, thereby avoiding too many sweet and sticky foodstuffs. Particular attention should be given to children, and oral hygiene, as well as healthy lifestyle habits, should be implemented in the educational curriculum so that from an early age, one is made aware of these basics to have a healthier and brighter future generation. Policymakers should invest in the promotion and prevention of these issues so that the financial impact due to the treatment and rehabilitation of these diseases is less.

## Author Contributions

RG, DT, and JW conceived the study, analyzed the data, and drafted the manuscript. QL participated in the study design and helped refine the manuscript. All authors contributed to the study and have read and approved the final version.

## Conflict of Interest

The authors declare that the research was conducted in the absence of any commercial or financial relationships that could be construed as a potential conflict of interest.

## Supplementary Material

Supplementary Table 1 Newcastle-Ottawa quality assessment scale.
