# Supplementary material for: Periodontal Diseases and the Risk of Metabolic Syndrome: An Updated Systematic Review and Meta-Analysis
Source: Front Endocrinol (Lausanne). 2020 Jun 9;11:336. doi: 10.3389/fendo.2020.00336 (PMC7296056; doi:10.3389/fendo.2020.00336)
Supplement: Supplementary Table 1 — Newcastle-Ottawa quality assessment scale. [file Table_1.doc]

**Supplementary table 1. Newcastle-Ottawa quality assessment scale.**

| **STUDY (author, year, ref.)** | **Selection** | **Comparability** | **Outcome** |
| --- | --- | --- | --- |
| LI, 2007 (15) | ** | ** | *** |
| SHIMAZAKI, 2007 (16) | ** | ** | ** |
| D'AIUTO, 2008 (17) | **** | ** | ** |
| KHADER, 2008 (18) | *** | * | ** |
| LI, 2009 (19) | ** | * | ** |
| MORITA, 2009 (20) | ** | * | ** |
| KUSHIYAMA, 2009 (21) | * | ** | ** |
| ANDRIANKAJA, 2010 (22) | *** | ** | ** |
| NESBITT, 2010 (23) | ** | * | * |
| BENGUIGUI, 2010 (24) | **** | ** | ** |
| HAN, 2010 (25) | *** | ** | ** |
| TIMONEN, 2010 (26) | *** | * | * |
| ZHANG, 2010 (27) | ** | * | ** |
| BENSLEY, 2011 (28) | ** | * | ** |
| KWON, 2011 (29) | ** | ** | ** |
| CHEN, 2011 (30) | ** | ** | * |
| HAN, 2012 (10) | ** | ** | ** |
| FUKUI, 2012 (31) | *** | ** | *** |
| YU, 2012 (32) | ** | ** | ** |
| TU, 2013 (33) | ** | * | * |
| SORA, 2013 (34) | *** | ** | *** |
| FURATA, 2013 (35) | **** | * | ** |
| LAMONTE, 2014 (36) | **** | ** | ** |
| THANAKUN, 2014 (37) | ** | * | * |
| ALHABASHNEH, 2015 (38) | *** | ** | *** |
| MINAGAWA, 2015 (39) | *** | ** | ** |
| IWASAKI , 2015 (40) | ** | ** | ** |
| CHEN, 2016 (41) | *** | * | ** |
| JARAMILLO, 2016 (42) | *** | ** | ** |
| KUMAR, 2016 (43) | *** | ** | *** |
| GOMES- FILHO, 2016 (44) | *** | ** | *** |
| KAYE, 2016 (45) | *** | ** | ** |
| MUSSKOPF, 2016 (46) | *** | ** | ** |
| WU, 2016 (47) | ** | ** | ** |
| ZUK, 2017 (12) | *** | ** | ** |
| KIKUI, 2017 (48) | *** | ** | ** |
| ZHANG, 2017 (49) | ** | ** | ** |
| KIM, 2018 (50) | *** | ** | ** |
| PHAM, 2018 (51)  KOO, 2018 (52) | ***  ** | **  ** | **  ** |
| NASCIMENTO, 2018 (53) | *** | ** | *** |
| ABDALLA-ASLAN, 2019 (6) | *** | ** | *** |
| KIM,2019 (54) | *** | ** | ** |
